# Intelligent Algorithm-Based MRI Image Features for Evaluating the Effect of Nursing on Recovery of the Neurological Function of Patients with Acute Stroke

**DOI:** 10.1155/2022/3936655

**Published:** 2022-05-31

**Authors:** Ding Wang, Jingwei Dai

**Affiliations:** ^1^Department of Neurosurgery, Shengjing Hospital of China Medical University, Liaoning 110000, Shenyang, China; ^2^Third Department of Neurology Ward, Shengjing Hospital Affiliated to China Medical University, Liaoning 110000, Shenyang, China

## Abstract

The aim of this study is to analyze the application of early rehabilitation nursing in nursing intervention of neurological impairment among patients with acute ischemic stroke. 116 patients with acute ischemic stroke were selected as the research subjects in this paper. The patients were divided into 58 experimental (early rehabilitation care) and 58 control (routine rehabilitation care) groups according to the difference of care protocols, all of which were performed magnetic resonance imaging on. An image resolution reconstruction algorithm on the basis of deep convolutional neural network is proposed for MRI image processing. The results show that peak signal to noise ratio (PSNR) and structural similarity index measure (SSIM) of the included algorithm were remarkably greater than those of compressed sensing (CS) algorithm and nonlocal similarity and block low rank prior-based NSBL algorithm. Running time was shorter than that of the latter two algorithms (*P* < 0.05). The neurological impairment scores of patients in the experimental group 3 and 5 weeks after treatment were obviously lower than those of patients in the control group (*P* < 0.05). The Barthel indexes of patients in the experimental group 3 and 5 weeks after treatment were obviously higher than those of patients in the control group (*P* < 0.05). FugI-Meyer assessment (FMA) and Disability of Arm-Shoulder-Hand (DASH) scores of patients in the experimental group 3 and 5 weeks after treatment were obviously lower than those of patients in control group (*P* < 0.05). The results show that the deep learning algorithm for MRI image processing performance is better than the traditional algorithm. It not only improves the image quality but also improves the processing efficiency. Early rehabilitation nursing and routine rehabilitation nursing can effectively improve the neurological deficit symptoms, limb motor function, and daily living ability of patients with acute ischemic stroke, and the effect of early rehabilitation nursing is the best.

## 1. Introduction

Acute stroke is due to various reasons that lead to blood supply disorders in local brain tissue, leading to ischemia and hypoxia in brain tissue and necrosis, resulting in corresponding symptoms and signs. It is a disease with high incidence, high recurrence rate, high death rate, high disability rate, and many complications. The common symptoms include weakness, numbness, or paralysis on one side or upper and lower limbs, weakness, numbness or paralysis on the face, blurred vision on one or both eyes, difficulty in language expression, dizziness, imbalance, and headache [1–3]. After treatment, about 70% of stroke patients have varying degrees of dysfunction. The severity of dysfunction depends on the damage to the brain structure [4, 5]. Clinical treatment of stroke mainly includes intravenous thrombolysis, vascular interventional therapy, antiplatelet anticoagulation and expansion, and improvement of cerebral vascular circulation. Conservative treatment and surgical treatment are also considered for acute stroke [6]. Statistics show that China has become one of the countries with the highest incidence of stroke. Its annual economic losses have reached more than 40 billion yuan [7]. Each year, the number of new patients reaches more than 2 million, of which ischemic stroke accounts for 70%. About 70%–80% of ischemic stroke patients lead to disability and cannot live independently [8]. With the aging of population, the development of the economic level and the change of lifestyle, the intervention of early nursing and rehabilitation therapy for patients with ischemic stroke is particularly important, so it is necessary to discuss.

Neuroimaging plays an important role in acute stroke. For example, to determine or exclude the diagnosis of cerebrovascular disease (ischemic or hemorrhagic) and to provide evidence of possible stroke pathogenesis (embolism, hemodynamics, etc.). Through the size and location of the lesion and vascular state, we provide important information about the prognosis and so on [9–11]. CT examination can be used as an accurate screening method. The differential point is that clinicians collect medical history and preliminarily determine that patients are involved in stroke after physical examination. Therefore, head CT is used in the first screening, which has the advantages of short time consumption and low cost [12]. MRI is superior to CT in differentiating acute ischemic stroke. The latest TTA report of the American Society of Neurology points out that DWI is determined to be useful for the diagnosis of acute ischemic stroke within 12 h of onset and should be more useful than nonenhanced CT [13, 14]. In addition, multiparameter MRI may prolong the treatment time window. The basis is the understanding of individual pathophysiological processes. MRI is used to identify patients most likely to benefit from treatment (those with ischemic penumbra or risk tissue) and those most likely to have complications (such as hemorrhagic transformation, malignant infarction) [15]. In clinical practice, due to the limitation of acquisition time, patient comfort, image signal-to-noise ratio, and other factors, it is often difficult to obtain ideal high-resolution magnetic resonance images. It is easy to affect the doctor's objective judgment [16]. At present, with the rapid development of deep learning, deep networks for image super-resolution problems emerge endlessly and have achieved very good results. Super-resolution (SR) reconstruction technology can reconstruct images with high resolution from low-resolution images, which has broad application prospects in the field of magnetic resonance. However, the traditional SR method belongs to supervised learning, which requires specific training data for image reconstruction with poor reconstruction effects.

In summary, the nursing treatment of neurological deficit symptoms in stroke patients still needs further discussion. This study selects 116 patients with acute ischemic stroke as the research subject. According to different nursing schemes, the patients were divided into 58 cases of experimental group (early rehabilitation nursing) and 58 cases of control group (routine rehabilitation nursing). All patients underwent MRI examination. An image resolution reconstruction algorithm on the basis of deep learning is proposed for MRI image processing. The therapeutic effect of early nursing rehabilitation on patients was comprehensively evaluated by comparing the neurological deficit symptoms, limb motor function, and daily living ability scores of the two groups before and after treatment.

## 2. Materials and Methods

### 2.1. Research Subjects

In this study, 116 patients with acute ischemic stroke admitted to hospital from June 1, 2019 to February 1, 2021 were selected as subjects. There were 72 males and 44 females aged 48–71 years. This study has been approved by the ethics committee of hospital. The family members of the patients signed the informed consent.

#### 2.1.1. Inclusion Criteria

The inclusion criteria were as follows: (1) to meet the diagnostic criteria of cerebral infarction determined by the Fourth National Cerebrovascular Disease Conference in 1995. (2) Patients who have signed the informed consent. (3) Patients with lateral limb dysfunction. (4) Patients with limb muscle strength less than or equal to grade IV. (5) Patients with stable life weight. (6) Patients those who were willing to participate in rehabilitation training.

#### 2.1.2. Exclusion Criteria

The exclusion criteria were as follows: (1) Patients with subarachnoid hemorrhage. (2) Patients with intracranial venous thrombosis. (3) Patients with severe pulmonary infection. (4) Patients with liver and kidney diseases, heart diseases, and other important organ damage. (5) Patients with limb muscle strength less than or equal to grade IV. (6) Patients with cognitive impairment. (7) Patients with neurological or musculoskeletal diseases affecting functional recovery.

### 2.2. Case Grouping and Nursing

According to different nursing schemes, the patients were divided into 58 cases of the experimental group (early rehabilitation nursing) and 58 cases of the control group (routine rehabilitation nursing).

Two groups of patients were responsible for rehabilitation training. Training began after the condition was stable and neurological symptoms were stable. Specific steps were as follows: (1) Teaching patients to make limbs in functional position. Family members and nursing staff adopted the correct posture for counseling: affected side position > healthy side position > supine position. Guide the patient to turn over every 2 hours and take back count. Avoid forming abnormal patterns of upper limb flexion, lower limb extension, and foot sag varus. (2) The hands and fingers crossed and held each other. The thumbs of the affected side were placed above, and the healthy limbs were used to drive the diseased limbs. Then, slowly put down the arms and placed in front of the abdomen. Upper limb joint activities include finger nose, lateral lifting, lower limb flexion brace bed hip lifting, namely, bridge movement. (3) Patients were encouraged to sit for training as early as possible after their condition stabilized. According to the patient' s muscle strength, patients should take the initiative as much as possible. Methods of family protection: the patient was moved laterally to the bedside, and the affected leg was hooked with a healthy leg to move out of the bed, making the affected knee buckled to the bedside. Then, the body rotates to the affected side, and the healthy hand crosses the body. At the same time, the healthy arm strength pushed the bed to sit up. When the torso had certain control ability, it can gradually let the patient use the healthy hand to assist the patient to picked up or dropped the object. Then, gradually put out to increase the difficulty of sitting training. (4) Lower limb muscle strength grade 3 and above for standing training. When standing training, the therapist's protection method was as follows: the upper limbs forward, head and trunk forward, the center of gravity moved between the feet. Then, raise the patient's hips and knees were gradually stretched and made to stand up. The patient held the parallel bars by hand, gradually increased the time and tried to shift the center of gravity to the affected side. Moreover, gradually increased the standing balance training. (5) With the increase of lower limb weight-bearing muscle strength, walking training can be gradually carried out. Then, gradual transition to across different obstacles and stairs walking training was given. It mainly increases brain blood supply, activates brain cells, and improves local muscle spasm, twitch, and incomplete paralysis so as to improve muscle strength and motor function. The above rehabilitation exercise treatment was carried out every 20–30 min, 2-3 times a day, 4-5 days a week. Rehabilitation exercise therapy lasted 3 months.

Both groups were given drug treatment at the same time. Drug therapy was conducted in accordance with acute ischemic cerebrovascular disease diagnosis and treatment guidelines. Basic medications include aspirin, ginkgo dipyridamole, and edaravone.

### 2.3. MRI Examination

The patients were examined by 3.0 T superconducting magnetic resonance scanning, and the 12 - channel phased array head coil was used. Gradient echo sequence of magnetization preparation, scanning parameters were as follows: TE 900 ms, TR 2,200 ms, FOV 256 × 256 × 150, 1 mm voxel collection. Planar echo imaging sequence, scanning parameters were as follows: TE 40 ms, TR 3,000 ms, FOV 215 × 215, 2 mm voxel acquisition, inversion angle 90°, scanning time 320 s. Finally, the obtained image data was input to the workstation for enhancement processing.

### 2.4. Deep Convolutional Neural Network (CNN)-Based Image Resolution Reconstruction Algorithm

**Figure 1:**
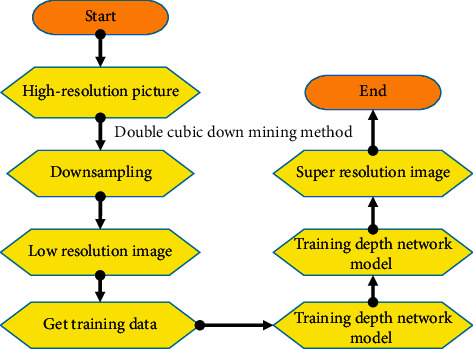
Conventional super-resolution image reconstruction method.

With the rapid development of deep learning, deep networks for image super-resolution problems emerge in endlessly and have achieved very good results. However, most super-resolution methods on the basis of deep learning belong to supervised learning, which limits the reconstruction process to specific training data. The basic framework of these methods is illustrated in Figure 1. The specific downsampling method was used to process the high-resolution images to obtain the corresponding low-resolution image, and then the corresponding training data were collected. A deep network model was trained. Then, the low-resolution image to be tested was input into the model to obtain the reconstructed super-resolution image.

The purpose of super-resolution reconstruction is to recover high-resolution images from low-resolution images. Set the low-resolution image to *P*, which can be expressed as follows:

(1) $$\begin{array}{c} P_{x}=C\left(P_{y};\theta \right). \end{array}$$

where *C* represents the degradation process function, *P*_*y*_ represents the high-resolution image, *θ* represents the degradation process parameters. Then, the downsampling operation can be expressed as follows:

(2) $$\begin{array}{c} C\left(P_{y};\theta \right)=\left(P_{y}\right){↓}_{r}, \end{array}$$

↓_*r*_ represents the downsampling operation of *r*, and {*r*} ∈ *θ*. In general, low-resolution images are obtained by bicubic anti-aliasing reduction of high-resolution images, so the degradation process can also be expressed as follows:

(3) $$\begin{array}{c} C\left(P_{y};\theta \right)=\left(P_{y}⊗\beta \right){↓}_{r}+u_{\tau }, \end{array}$$

where *β* represents fuzzy kernel, *P*_*y*_ ⊗ *β* represents convolution operation between high-resolution image and fuzzy kernel, and *u*_*τ*_ represents Gaussian white noise. The objective function of super-resolution reconstruction can be expressed as follows:

(4) $$\begin{array}{c} \alpha *=\underset{\alpha }{\arg\min\text{ loss}\left(P_{y}*,P_{y}\right)}+\eta \Phi \left(\alpha \right), \end{array}$$

where loss(*P*_*y*_*∗*, *P*_*y*_) represents the loss function and Φ(*α*) represents the regularization term. The above supervised image super-resolution algorithm can obtain good results. But a lot of paired training data are needed to train the network. For magnetic resonance imaging, which is limited by hardware conditions, it is difficult to obtain such data sets. Therefore, this paper regards super-resolution image reconstruction as an ill-posed inverse problem, that is, low-resolution images correspond to multiple different high-resolution images. Considering the actual operation process, image quality will be affected by many aspects. For example, the motion artifacts caused by the movement of the subjects in the data acquisition process, the black band artifacts caused by the inhomogeneity of the magnetic field, and the noise caused by the current fluctuation in the circuit, etc. So, introducing these factors into image degradation model can be expressed as follows:

(5) $$\begin{array}{c} P_{L}=C_{\text{down}}T_{H}+\chi , \end{array}$$

where *P*_*L*_ represents a low-resolution image, *T* represents degradation function, *C*_down_ represents the downsampling operator, and *χ* represents noise.

In magnetic resonance imaging, the degradation function can be seen as a part of the k-space center of the original high-resolution magnetic resonance image that intercepts the corresponding proportion. Therefore, a low-resolution magnetic resonance image is given to recover the image version as similar as the real collected high-resolution image. This study reconstructs super-resolution images by minimizing the following loss functions:

(6) $$\begin{array}{c} P_{H}^{*}=\arg\min P_{H}\left\|P_{L}-C_{\text{down}}T_{H}\right\|, \end{array}$$

where *P*_*H*_^*∗*^ represents the reconstruction of high-resolution images. The above formulation often leads to an unstable solution. So, it is necessary to give a suitable regularization term to limit the range of solution space, then the model can be updated to as follows:

(7) $$\begin{array}{c} P_{H}^{*}=\arg\min P_{H}{\left\|P_{L}-C_{\text{down}}T_{H}\right\|}^{2}+\eta {\left\|P_{H}-l_{0}e^{-\left(\text{TIME}/P_{\sigma }\right)}\right\|}^{2}. \end{array}$$

TIME represents the spin locking time, *l*_0_ represents the magnetization in equilibrium state, and *P*_*σ*_ represents the parameters to be fitted. The weighted images collected at different spin locking times were fitted by the second exponential decay model. argmin*P*_*H*_‖*P*_*L*_ − *C*_down_*T*_*H*_‖^2^ ensures data consistency. ‖*P*_*H*_ − *l*_0_*e*^−(TIME/*P*_*ω*_)^‖^2^ ensures model consistency. Since the purpose of network learning is to learn the mapping from low-resolution images to high-resolution images, the above can also be expressed as follows:

(8) $$\begin{array}{c} P_{H}^{*}=\arg\min P_{H}{\left\|P_{L}-{\left(C_{\text{down}}T\right)}^{-1}P_{L}\right\|}^{2}+\eta {\left\|P_{H}-l_{0}e^{-\left(\text{TIME}/P_{\omega }\right)}\right\|}^{2}P_{\sigma }, \end{array}$$

where (*C*_down_*T*)^−1^ represents the combination of the recovery operator and the sampling on the image.

### 2.5. Image Quality Assessment Indicators

In this study, peak signal-to-noise ratio (PSNR) [17] and structural similarity (SSIM) [18] are used as two parameters to evaluate image quality.

PSNR can be defined by the maximum pixel value and mean square error between images, which can be expressed as follows:

(9) $$\begin{array}{c} PSNR=10*\;\log_{10}\left(\frac{K^{2}}{\left(1/M\right)\left(\sum_{i=1}^{M}\left(P\left(i\right)-P^{*}{\left(i\right)}^{2}\right)\right)}\right). \end{array}$$

where *M* represents pixels, *P*^*∗*^ represents the reconstructed image, and *P* represents the original image.

SSIM is used to measure the structural similarity between images. The evaluation criteria are based on the brightness, contrast, and structure of the image, which can be expressed as follows:

(10) $$\begin{array}{c} \text{SSIM}={\left[R_{1}\left(P,P^{*}\right)\right]}^{a}{\left[R_{2}\left(P,P^{*}\right)\right]}^{b}{\left[R_{3}\left(P,P^{*}\right)\right]}^{c},\\ R_{1}\left(P,P^{*}\right)=\frac{\left(2\upsilon_{P}\upsilon_{P*}+R_{0}\right)}{\left(\upsilon_{P}^{2}+\upsilon_{P*}^{2}+R_{0}\right)},\\ R_{2}\left(P,P^{*}\right)=\frac{\left(2\varsigma_{P}\varsigma_{P*}+R_{01}\right)}{\left(\varsigma_{P}^{2}+\varsigma_{P*}^{2}+R_{01}\right)}, \end{array}$$

where *υ*_*P*_ represents image brightness and *ς*_*P*_ represents contrast ratio.

### 2.6. Observation Indicators

The general data (age, gender, course of disease) of the two groups were recorded. The functional indexes before treatment, 3 weeks after treatment, and 5 weeks after treatment were recorded. The method suggested by National Institutes of Health Stroke (NIHSS) was used to assess the degree of neurological deficits. Activities of daily living were assessed by the modified Barthel Index (MBI). FugI-Meyer Assessment (FMA) was used to assess limb motor function. The DASH (disability of Arm-Shoulder-Hand) scale was used to score the patients before and after treatment [19].

### 2.7. Statistical Methods

The data in this study were analyzed by SPSS19.0. The measurement data are expressed as mean ± standard deviation ( ± *s*). Count data are expressed as percentage (%). One-way ANOVA was used for pairwise comparison. The difference was statistically significant with *P* < 0.05.

## 3. Results

### 3.1. Algorithm Performance Display

**Figure 2:**
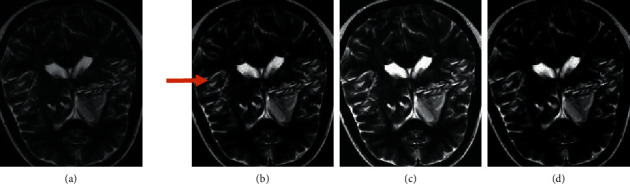
Performance of the algorithm. (a) is the original image, (b) is the image reconstruction for CS algorithm, (c) is the NSBL algorithm to reconstruct the image, and (d) is the reconstruction of images for the algorithm.

The MRI image reconstruction algorithm based on compressed sensing (CS) [20], the MRI image reconstruction algorithm based on nonlocal similarity and block low rank prior (NSBL) [21] are introduced, and compared with the algorithms designed in this study. It can be seen from the actual MRI image reconstruction (Figure 2) that the overall quality of the original image is poor, there are more artifacts and noises, and the resolution is low, and the clarity is obviously not enough. After the three algorithms, the quality has been significantly improved. Among them, the algorithm in this study has the best re-effect on MRI images, the clarity has been significantly improved, and the artifacts and noise have been greatly reduced.

**Figure 3:**
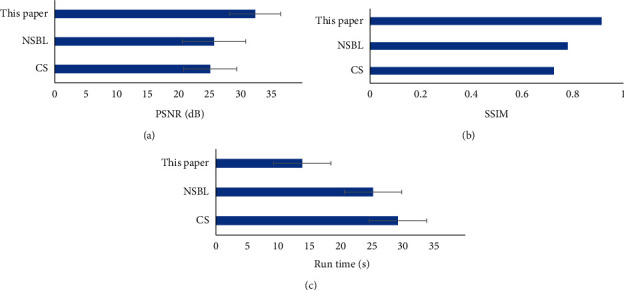
Quantitative analysis of algorithm performance. (a) is PSNR; (b) is SSIM; and (c) is the running time. ^*∗*^ Compared with the proposed algorithm, *P* < 0.05.

From the quantitative data results (Figure 3), the PSNR and SSIM of the proposed algorithm are significantly greater than those of the CS algorithm and the NSBL algorithm. The difference was statistically significant (*P* < 0.05). The running time of the proposed algorithm is significantly shorter than that of the CS algorithm and NSBL algorithm. The difference was statistically significant (*P* < 0.05).

### 3.2. Comparison of Basic Data between the Two Groups of Patients

**Figure 4:**
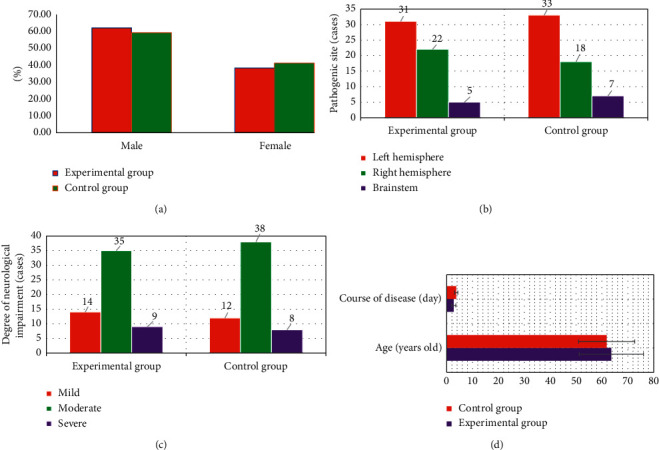
Comparisons of the data of the two groups. (a) is gender; (b) is the location of the disease; (c) is the degree of neurological deficit; and (d) is the age and course of disease. ^*∗*^ Compared with the proposed algorithm, *P* < 0.05.

The basic data of the two groups were compared (Figure 4). There was no significant difference in age, gender, course of disease, location of disease (left hemisphere, right hemisphere, brainstem), and degree of nerve deficit (mild, moderate, and severe) between the two groups (*P* > 0.05).

### 3.3. MRI Imaging Data of Patients

**Figure 5:**
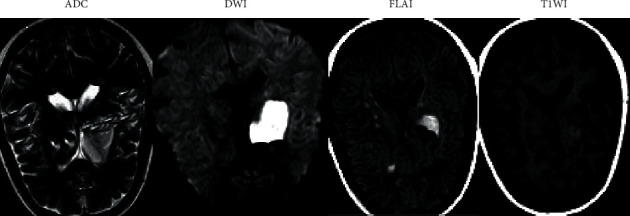
MRI images of early acute ischemic stroke.

As illustrated in Figure 5, the patient was a 59-year-old woman. The patient was treated for weakness and rigidity of the right lower limb. ADC map showed the low-signal region at the right parietal-occipital junction, FLAIR image showed a high signal in the corresponding region, T1WI showed a low signal in the corresponding region, and T1-enhanced scan showed brain parenchyma enhancement in the affected region.

**Figure 6:**
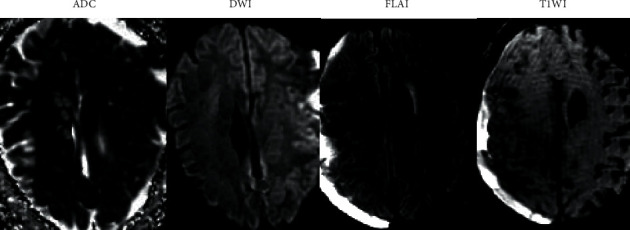
MRI images of early ischemic stroke in the subacute phase.

Figure 6 reveals that the patient is a 70-year-old male. DWI displayed the low-signal region of the right occipital lobe, and the high signal edge was visible around it. This may be caused by the T2 penetration effect. The ADC map revealed a high signal in the corresponding region, SWI revealed the results of right occipital lobe hemorrhage, and T2WI revealed a high signal in the corresponding region of right occipital lobe.

### 3.4. Neurological Deficit Scores of the Two Groups before and after Treatment

**Figure 7:**
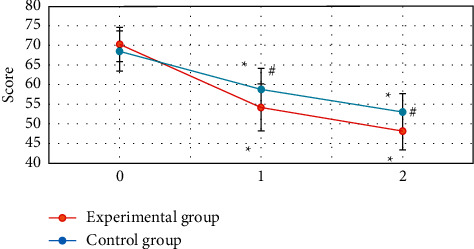
Two groups of patients before and after treatment neurological deficit score 0 is before treatment; 1 is three weeks after treatment; and 2 is five weeks after treatment. ^#^Compared with that before treatment, *P* < 0.05; #compared with the experimental group, *P* < 0.05.

Figure 7 shows that the difference in neurological deficit scores between the two groups before treatment was not statistically significant (*P* > 0.05). The neurological deficit scores of the two groups at 3 and 5 weeks after treatment were significantly lower than those before treatment, and the difference was statistically significant (*P* < 0.05). The neurological deficit score of the experimental group was significantly lower than that of the control group at 3 and 5 weeks after treatment, and the difference was statistically significant (*P* < 0.05).

### 3.5. Comparison of the Barthel Index before and after Treatment between the Two Groups

**Figure 8:**
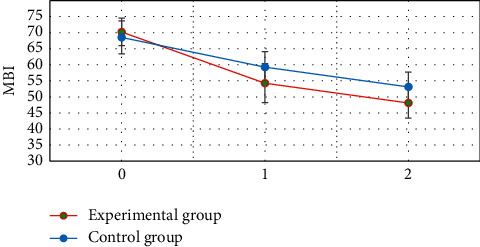
Barthel index of two groups before and after treatment. 0 is before treatment; 1 is three weeks after treatment; and 2 is 5 weeks after treatment. ^*∗*^ Compared with that before treatment, *P* < 0.05; ^#^compared with the experimental group, *P* < 0.05.

Figure 8 shows that the difference in the Barthel index between the two groups before treatment was not statistically significant (*P* > 0.05). The Barthel index of the two groups at 3 and 5 weeks after treatment was significantly higher than that before treatment, and the difference was statistically significant (*P* < 0.05). The Barthel index of the experimental group was significantly higher than that of the control group at 3 and 5 weeks after treatment, and the difference was statistically significant (*P* < 0.05).

### 3.6. Comparison of Motor Function Scores between the Two Groups before and after Treatment

**Figure 9:**
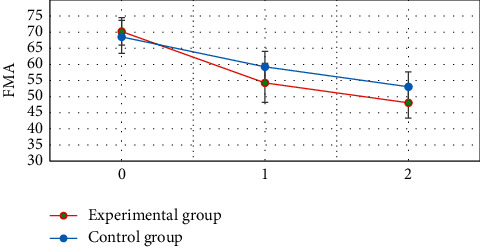
FMA scores of two groups before and after treatment. 0 is before treatment. 1 is three weeks after treatment. 2 is 5 weeks after treatment. ^*∗*^ Compared with that before treatment, *P* < 0.05; ^#^compared with the experimental group, *P* < 0.05.

Figure 9 shows that the FMA difference between the two groups before treatment was not statistically significant (*P* > 0.05). FMA of the two groups at 3 and 5 weeks after treatment was significantly lower than that before treatment, and the difference was statistically significant (*P* < 0.05). FMA of the experimental group at 3 and 5 weeks after treatment was significantly lower than that of the control group, and the difference was statistically significant (*P* < 0.05).

### 3.7. Comparison of DASH between the Two Groups before and after Treatment

**Figure 10:**
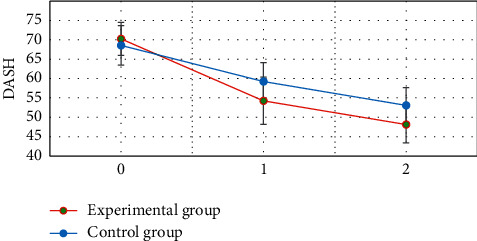
DASH scores of two groups before and after treatment. 0 is before treatment. 1 is three weeks after treatment. 2 is 5 weeks after treatment. ^*∗*^ Compared with that before treatment, *P* < 0.05; ^#^compared with the experimental group, *P* < 0.05.

Figure 10 shows that the difference in DASH between the two groups before treatment was not statistically significant (*P* > 0.05). The DASH of the two groups at 3 and 5 weeks after treatment was significantly lower than that before treatment, and the difference was statistically significant (*P* < 0.05). The DASH of the experimental group was significantly lower than that of the control group at 3 and 5 weeks after treatment, and the difference was statistically significant (*P* < 0.05).

## 4. Discussion

Stroke is caused by a sudden rupture of blood vessels in the brain bleeding or cerebral vascular embolism caused by blood that cannot flow into the brain due to brain tissue damage. Stroke can lead to cerebral ischemia, hypoxia, necrosis, and other pathological changes, which lead to neurological deficits and other neurological symptoms. Its severity will directly affect the prognosis of patients, which is one of the topics of widespread concern in clinical practice [22]. Therefore, 116 patients with acute ischemic stroke were selected as the research subjects in this study. According to different nursing schemes, the patients were divided into 58 cases of the experimental group (early rehabilitation nursing) and 58 cases of the control group (routine rehabilitation nursing). The patients were examined by Avanto3.0 T superconducting magnetic resonance scanning in Siemens, Germany. The PSNR and SSIM of the proposed algorithm are significantly larger than those of the CS algorithm and NSBL algorithm. The running time was significantly shorter than that of the CS algorithm and NSBL algorithm, and the difference was statistically significant (*P* < 0.05). This is similar to the research results of Kopczak et al. (2020) [23]. It shows that the deep learning algorithm constructed in this paper has better processing performance than the traditional algorithm for MRI images, which not only improves the image quality but also improves the processing efficiency. The basic data of the two groups were compared, and it can be seen that there was no statistically significant difference in age, gender, course of disease, location of disease (left hemisphere, right hemisphere, and brainstem), and degree of nerve deficit (mild, moderate, and severe) between the two groups (*P* > 0.05). This provides feasibility for subsequent research.

The neurological deficit scores of the two groups at 3 and 5 weeks after treatment were significantly lower than those before treatment. The neurological deficit scores of the experimental group at 3 and 5 weeks after treatment were significantly lower than those of the control group, and the difference was statistically significant (*P* < 0.05). This is consistent with the research results of Lu et al. (2020) [24]. It indicated that early rehabilitation nursing and routine rehabilitation nursing could effectively improve the neurological deficit symptoms of patients with acute ischemic stroke, and the effect of early rehabilitation nursing was better. The Barthel index of the two groups at 3 and 5 weeks after treatment was significantly higher than that before treatment. The Barthel index of the experimental group was significantly higher than that of the control group at 3 and 5 weeks after treatment, and the difference was statistically significant (*P* < 0.05). This also reveals that early rehabilitation nursing can more effectively improve the daily living ability of stroke patients [25]. The FMA and DASH scores of the two groups at 3 and 5 weeks after treatment were significantly lower than those before treatment. The FMA and DASH scores of the experimental group were significantly lower than those of the control group at 3 and 5 weeks after treatment, and the difference was statistically significant (*P* < 0.05). This reveals that early rehabilitation nursing can more effectively improve the limb motor ability of stroke patients.

## 5. Conclusion

In this study, 116 patients with acute ischemic stroke were selected as subjects. According to different nursing schemes, the patients were divided into 58 cases of the experimental group (early rehabilitation nursing) and 58 cases of the control group (routine rehabilitation nursing). Patients were examined by magnetic resonance imaging on the basis of deep learning algorithm. The results show that the deep learning algorithm constructed in this paper has better processing performance than the traditional algorithm for MRI images, which not only improves the image quality but also improves the processing efficiency. Early rehabilitation nursing and routine rehabilitation nursing can effectively improve the neurological deficit symptoms, limb motor function, and daily living ability of patients with acute ischemic stroke, and the effect of early rehabilitation nursing is the best. However, only 116 patients with acute cerebral stroke were included. The sample size was small with single source. Besides, there was apparent regional limitations. Therefore, a wider range of cases needed to be included in subsequent studies to further discuss clinical nursing intervention paths in ischemic stroke. In summary, this study provides a theoretical support for the clinical nursing treatment of patients with ischemic stroke.

## Data Availability

The data used to support the findings of this study are available from the corresponding author upon request.
